# Dimeric RNA Recognition Regulates HIV-1 Genome Packaging

**DOI:** 10.1371/journal.ppat.1003249

**Published:** 2013-03-21

**Authors:** Olga A. Nikolaitchik, Kari A. Dilley, William Fu, Robert J. Gorelick, S.-H. Sheldon Tai, Ferri Soheilian, Roger G. Ptak, Kunio Nagashima, Vinay K. Pathak, Wei-Shau Hu

**Affiliations:** 1 HIV Drug Resistance Program, National Cancer Institute, Frederick, Maryland, United States of America; 2 Southern Research Institute, Frederick, Maryland, United States of America; 3 AIDS and Cancer Virology Program, SAIC-Frederick Inc., Frederick National Laboratory for Cancer Research, Frederick, Maryland, United States of America; 4 Electron Microscopy Laboratory, SAIC-Frederick Inc., Frederick National Laboratory for Cancer Research, Frederick, Maryland, United States of America; Universitätklinikum Heidelberg, Germany

## Abstract

How retroviruses regulate the amount of RNA genome packaged into each virion has remained a long-standing question. Our previous study showed that most HIV-1 particles contain two copies of viral RNA, indicating that the number of genomes packaged is tightly regulated. In this report, we examine the mechanism that controls the number of RNA genomes encapsidated into HIV-1 particles. We hypothesize that HIV-1 regulates genome packaging by either the mass or copy number of the viral RNA. These two distinct mechanisms predict different outcomes when the genome size deviates significantly from that of wild type. Regulation by RNA mass would result in multiple copies of a small genome or one copy of a large genome being packaged, whereas regulation by copy number would result in two copies of a genome being packaged independent of size. To distinguish between these two hypotheses, we examined the packaging of viral RNA that was larger (≈17 kb) or smaller (≈3 kb) than that of wild-type HIV-1 (≈9 kb) and found that most particles packaged two copies of the viral genome regardless of whether they were 17 kb or 3 kb. Therefore, HIV-1 regulates RNA genome encapsidation not by the mass of RNA but by packaging two copies of RNA. To further explore the mechanism that governs this regulation, we examined the packaging of viral RNAs containing two packaging signals that can form intermolecular dimers or intramolecular dimers (self-dimers) and found that one self-dimer is packaged. Therefore, HIV-1 recognizes one dimeric RNA instead of two copies of RNA. Our findings reveal that dimeric RNA recognition is the key mechanism that regulates HIV-1 genome encapsidation and provide insights into a critical step in the generation of infectious viruses.

## Author Summary

Viruses must package their genomes in particles to pass their genetic information to the next generation. Although many aspects of RNA packaging are well-studied, how retroviruses regulate the number of genomes in the particle is currently unknown. Based on the dimeric nature of retroviral genomes in particles, it was often assumed that two copies of RNA were packaged into one particle. This assumption was validated recently when we demonstrated that most HIV-1 particles contain two copies of viral RNA, which revealed that the number of genomes packaged is tightly controlled. In this report, we examined the mechanism that regulates the amount of RNAs encapsidated into HIV-1 particles. Our results showed that RNA packaging is not regulated by the mass of the viral RNA as two copies of small or large genomes are packaged. However, packaging of two copies of RNA can be perturbed; HIV-1 can encapsidate one copy of its genome when the RNA contains two packaging/dimerization signals that allow for intramolecular dimer (self-dimer) formation. These studies revealed that HIV-1 regulates genome packaging by recognizing the dimeric RNA structure, and suggest that the interaction of viral protein Gag and dimeric RNA serves as the nucleation point of virus assembly.

## Introduction

Retroviruses are RNA viruses that replicate through a DNA phase, in which viral DNA is integrated into the host genome to form a provirus [1]. Retroviral genomes in virions are dimers, consisting of two copies of full-length, unspliced RNA, each of which encodes all of the genetic information needed for virus replication [2], [3], [4], [5], [6]. Packaging of the retroviral genome is mediated by interactions between the viral structural protein Gag and the *cis*-acting element(s), collectively called the packaging signal, in the viral RNA [7], [8], [9], [10], [11], [12], [13], [14]. In some retroviruses, such as HIV-1, RNA partner selection and the initiation of the dimerization process occur in the cytoplasm [15]; therefore, RNA dimerizes before encapsidation [16], [17]. A 6-nt sequence located at the 5′ untranslated region of the HIV-1 genome, termed the dimerization initiation signal (DIS), forms intermolecular base-pairs between two HIV-1 RNAs to initiate the dimerization process [13], [18], [19], [20], [21]. There are multiple DIS sequences in the circulating HIV-1 strains; two of the most common sequences are GCGCGC and GUGCAC [22], [23]. As base-pairing is involved in RNA partner selection for the initial dimerization process, the identity of the DIS sequence affects the ability of two HIV-1 RNAs derived from different proviruses to be copackaged together to form heterozygous particles [16].

Many aspects of retroviral RNA genome encapsidation, such as the *cis-* and *trans-*acting elements that mediate the specific packaging of the viral genome into particles, are well-studied. In contrast, other aspects of RNA packaging, such as how retroviruses regulate the number of genomes packaged into virions, are poorly understood. For example, it is not known whether the viral particle would package more than two copies of RNA from a viral genome that is much smaller than wild type. The possibility that multiple smaller RNA genomes are incorporated into one virion has been suggested previously [24]. However, the sensitivity of RNA detection method has made it difficult to confirm this suggestion. Similarly, it is also unclear whether retroviruses can efficiently package two copies of RNA that are much longer than their respective wild-type genomes. The lengths of the RNA genomes for most of the known avian and mammalian orthoretroviruses are close to 8–10 kb [25]. Additionally, the inability to efficiently encapsidate RNAs that are >2 kb larger than the wild-type genome led to the proposal of “packaging limit” of viral RNA [26], [27], [28]; this hypothesis implies that there may be physical barrier(s) to the encapsidation of larger RNA genomes. Other studies showed that vector RNAs much larger than viral genomes can be packaged into particles [29], [30]; however, it is not known whether one copy or two copies of vector RNAs were encapsidated into a particle.

By determining the viral RNA content of individual particles, our recent study demonstrated that most HIV-1 particles contain viral RNA; furthermore, two copies of RNA are packaged in each virion [16]. These observations indicate that the amount of HIV-1 RNA packaged into a particle is tightly regulated. We propose that HIV-1 RNA packaging is regulated by one of two mutually exclusive mechanisms: encapsidation may be regulated either by the total mass of viral RNA packaged or by the copy number of the packaged RNAs. These two hypotheses predict that viral RNA length will have different effects on packaging. The “RNA mass” hypothesis predicts that when the viral RNAs are smaller, more copies of RNA will be packaged; additionally, HIV-1 particle cannot accommodate two copies of viral RNAs much larger than those of “wild-type” virus. In contrast, the “copy number” hypothesis predicts that two copies of packagable viral RNAs will be encapsidated, independent of their size.

To test these two hypotheses, we examined packaging of HIV-1 genomes that are much larger or smaller than the wild-type 9-kb RNA by directly visualizing the viral RNA contents of individual particles. In this previously described single-virion analysis assay [16], HIV-1 genomes are engineered to contain stem-loop sequences recognized by either the coat protein of bacteriophage MS2 or the BglG protein from *Escherichia coli*, which are tagged with yellow fluorescent protein (YFP) and a red fluorescent protein, mCherry, respectively. Viral particles are visualized by fluorescence microscopy as a portion of the Gag proteins are tagged with cerulean fluorescent protein (CeFP), and the packaged viral RNAs are distinguished by their YFP or mCherry signals. In the current study, we modified this system to express RNA genomes that are ≈3 kb or nearly 17 kb so that we could examine the effects of viral RNA length on RNA packaging. Our results indicate that HIV-1 packages two copies of viral genome regardless of whether the RNA is ≈3 kb or ≈17 kb. We also found that HIV-1 can package one copy of the genome when the RNA contains two dimerization/packaging signals allowing for the formation of an intramolecular dimer (self-dimer). This observation indicates that the recognition of the dimeric RNA structure is a key element in the regulation of RNA encapsidation by HIV-1.

## Results

### HIV-1 Particles Can Package Viral Genomes Much Larger than 9 kb

**Figure 1:**
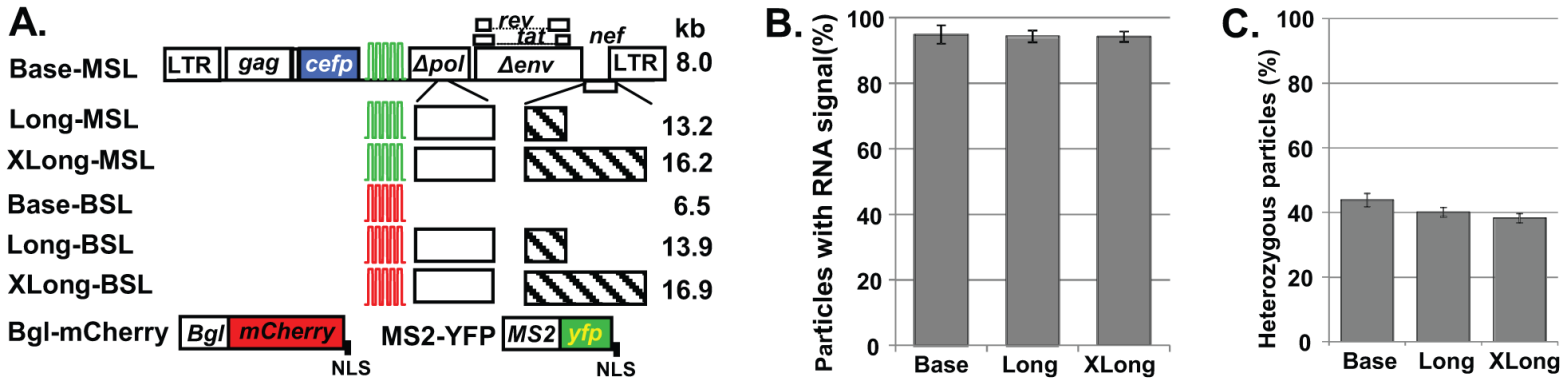
The ability of HIV-1 particles to package RNAs larger than 9 kb. (A) General structure of constructs. Green and red stem-loops represent binding sites for MS2 coat protein and BglG protein, respectively; white and hatched boxes denote HIV-1 and nonviral sequences, respectively; LTR, long terminal repeat; NLS, nuclear localization signal. (B) Packaging efficiencies of HIV-1 RNAs from Base, Long, and XLong constructs. Data shown are the mean ± standard deviation (SD) from ≥8 independent experiments. (C) Proportions of heterozygous particles generated by Base, Long, and XLong constructs. Data shown are the mean ± SD from ≥3 independent experiments.

We generated a series of constructs expressing larger RNA genomes based on the previously described HIV-1 constructs GagCeFP-MSSL and GagCeFP-BglSL [16], which are referred to as Base-MSL and Base-BSL, respectively (Figure 1A). Base-MSL and Base-BSL are derived from NL4-3 and contain all of the *cis*-acting elements required to express, export, and package HIV-1 RNA; they express functional Gag-CeFP, Tat, and Rev proteins, whereas the *pol*, *env*, *vif*, *vpr*, and *vpu* genes were inactivated by deletions. Additionally, Base-MSL and Base-BSL contain stem-loop sequences in the *pol* gene positions that are recognized by the coat protein of MS2 bacteriophage and the BglG protein of *E. coli*, respectively.

We inserted viral and nonviral sequences into Base-MSL and Base-BSL to generate the HIV-1 constructs Long-MSL and Long-BSL, respectively; the full-length viral RNAs expressed from these two constructs are 13–14 kb (Figure 1A). We then inserted additional nonviral sequences into Long-MSL and Long-BSL to generate XLong-MSL and XLong-BSL, respectively, which express 16–17-kb full-length viral RNAs. Although not shown, each Gag-CeFP-expressing construct has a corresponding construct that expresses untagged Gag; in each experiment, Gag-CeFP- and Gag-expressing constructs were cotransfected at equimolar ratios to preserve normal particle morphology [31].

The size of HIV-1 genomic RNA from the NL4-3 molecular clone is 9.1 kb. Previously we showed that genomic RNAs from our Base constructs, ranging from 6 to 8 kb in size, are efficiently packaged; RNA signals were detected in >90% of the viral particles [16]. To determine the efficiency with which HIV-1 packages RNAs larger than wild-type genomes, we cotransfected HIV-1 constructs expressing untagged and CeFP-tagged Gag along with MS2-YFP and Bgl-mCherry into 293T cells, collected viral particles, and analyzed them by fluorescence microscopy. In these experiments, the Gag signal is detected in the CeFP channel, whereas MS2 and BglG stem-loop-containing RNA signals are detected in the YFP and mCherry channels, respectively. The RNA-packaging efficiency is calculated as the percentage of CeFP^+^ particles that are positive for RNA signals (mCherry^+^ or YFP^+^). Our results showed that viral RNAs were packaged efficiently (>93%) in particles derived from Base, Long, and XLong constructs (≥4 independent experiments; >9,000 viral particles from each construct analyzed). Similar results were obtained from BglG and MS2 stem-loop-containing constructs (Table S1); for simplicity, these results are combined and shown in Figure 1B. These findings indicate that RNA genomes close to 17 kb can be packaged efficiently into HIV-1 particles.

### HIV-1 Packages Two Copies of Large RNAs

To determine whether viral particles generated by Long constructs encapsidate one or more genomic RNAs, we coexpressed Long-BSL and Long-MSL and performed single-virion analyses on the particles. If HIV-1 RNAs from two viruses are expressed at equal levels in the cells and assorted randomly before encapsidation, 50% of the viral population should be heterozygous particles that contain one RNA from each parent virus (Hardy-Weinberg equilibrium). In our previous experiments using the Base constructs, we found that ≈45% of the CeFP^+^ particles were YFP^+^mCherry^+^, which was very close to the predicted 50% heterozygous particles in the viral population [16].

Our analyses of the particles that were generated by coexpressing the Long-MSL and Long-BSL constructs showed that ≈40% of the CeFP^+^ particles were YFP^+^mCherry^+^; similarly, ≈38% of the CeFP^+^ particles that were generated by coexpressing XLong-MSL and XLong-BSL were YFP^+^mCherry^+^ (≥3 independent experiments; >20,000 particles from each pair of constructs analyzed; Figure 1C, Table S2). Compared with particles produced by the Base constructs (≈44%; Figure 1C, Table S2), virions derived from Long and XLong constructs generated heterozygous particles at efficiencies of 91% (40%/44%) and 86% (38%/44%). These results revealed that most of the particles derived from Long and XLong constructs contained two copies of HIV-1 RNAs.

### Biochemical Characterization Confirms the Encapsidation of Large Dimeric RNAs

**Figure 2:**
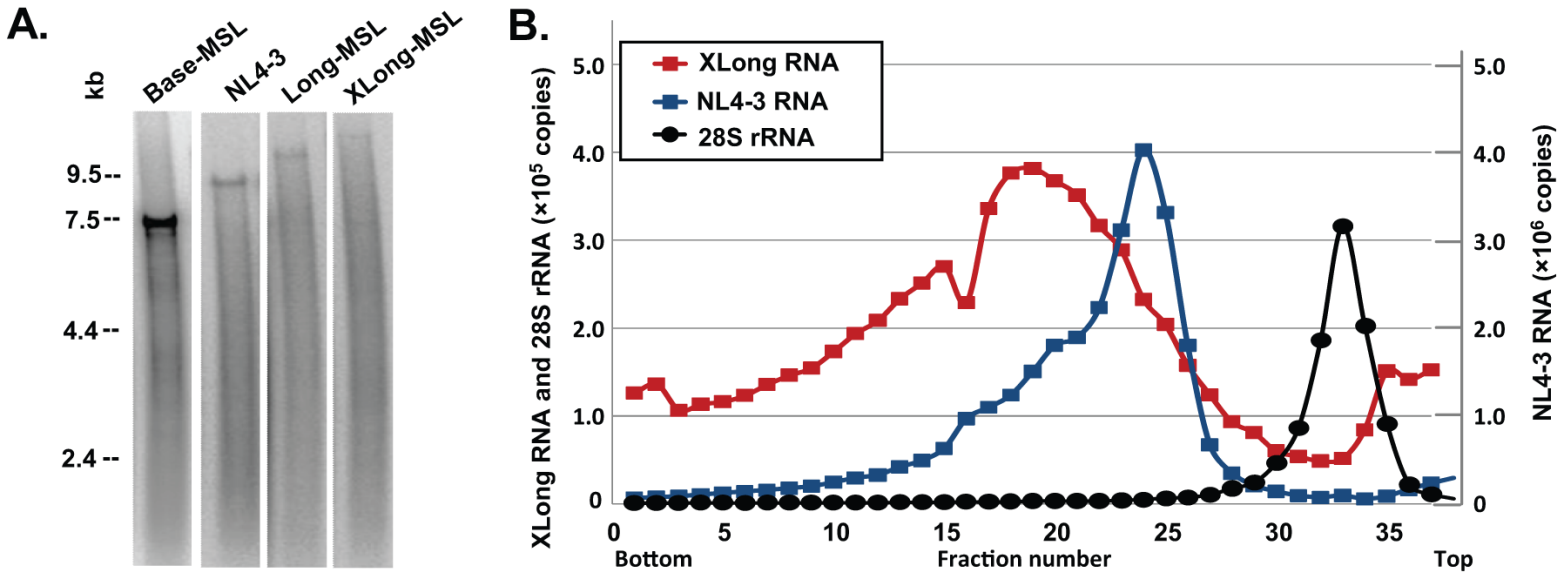
Biochemical analyses of HIV-1 virion RNAs. (A) Representative denaturing Northern analyses of virion RNAs. The four lanes shown are generated from the same gel and rearranged for clarity. (B) Representative analyses of velocity sedimentation of virion RNAs. The copy numbers of XLong and 28S rRNAs are shown in the *y* axis on the left, whereas the copy numbers of the NL4-3 RNA are shown in the *y* axis on the right.

In Base, Long, and XLong RNAs, the RNA-binding protein recognition sites are located in *pol*; therefore, only unspliced RNA should contain these sequences and be labeled with MS2-YFP or Bgl-mCherry. To confirm that the full-length RNAs were encapsidated, we first performed denaturing Northern analyses using virion RNA generated from NL4-3 as a control. As shown in a representative Northern blot (Figure 2A), full-length RNAs are evident in all four samples at the expected sizes; however, their abundance decreases with the size of the genome. This result is most likely caused by nicking of the RNA either in the particle or during preparation of the RNA samples. If the breakage of RNA is the same in a given length of RNA (for example, one break every 5 kb), then more of the smaller RNA (such as 7 kb) than the larger RNA (≈17 kb) can be expected to remain intact. It is worth noting that we did not observe distinct bands of short transcripts corresponding to spliced variants of the Long and XLong RNAs in these analyses.

To assess the size of the RNA dimer in the viral particles, we analyzed virion RNAs by velocity sedimentation through a sucrose gradient. We isolated RNAs from the viral particles derived from NL4-3 or XLong-BSL and loaded the RNA samples without heat treatment to preserve viral dimers on a 15%–30% sucrose gradient along with cellular RNAs isolated from uninfected 293T cells. After centrifugation, 37 fractions were collected, and the amounts of HIV-1 RNA and human 28S rRNAs were analyzed by quantitative real-time RT-PCR using primers and probes annealing to *gag* and 28S rRNAs, respectively. In these experiments, RNAs with higher molecular weights should sediment faster and migrate to fractions closer to the bottom of the tube. Results from a set of representative velocity sedimentation experiments are shown in Figure 2B. The NL4-3 RNA dimer (≈18 kb) migrates as a distinct population with the peak located at fraction 24, whereas the XLong RNA dimer migrates to fractions closer to the bottom of the tube with a peak at fraction 19. The 28S rRNAs (5 kb) from the uninfected 293T cells migrated slower than RNAs from NL4-3 or XLong and were detected in fractions closer to the top of the tubes with a peak at fraction 33. These results confirm that the dimeric RNA genomes packaged into viral particles from XLong constructs are larger than those from NL4-3.

### Long and XLong Constructs Generate Particles with Normal Immature Morphology

Results from our single-virion analyses revealed that most of the viral particles derived from Long and XLong constructs contained two copies of viral RNA genomes that were larger than 9 kb. To examine whether encapsidating larger RNA distorts virion morphology, we performed EM studies on various viral particles. The *pro* genes of Base, Long, and XLong constructs were deleted; therefore, we expected that these constructs would generate immature particles. As a control, we included viral particles produced from an NL4-3-derived construct (H0-PR*) that contains intact *gag-pol* with an inactivating mutation (D25N) in *pro*. Particles derived from all of these constructs exhibit similar immature morphology (Figure S1A); furthermore, these particles have similar sizes regardless of whether they were derived from Base, Long, or XLong constructs (>250 viral particles measured in each category; Figure S1B). These results indicate that packaging genomic RNAs almost twice as large as the wild-type, 9-kb RNA genome does not significantly alter the virion morphology; therefore, HIV-1 viral particles have the capacity to encapsidate two copies of genomes that are significantly larger than 9 kb.

### Short HIV-1 Genomic RNAs Are Packaged as Single Dimers

**Figure 3:**
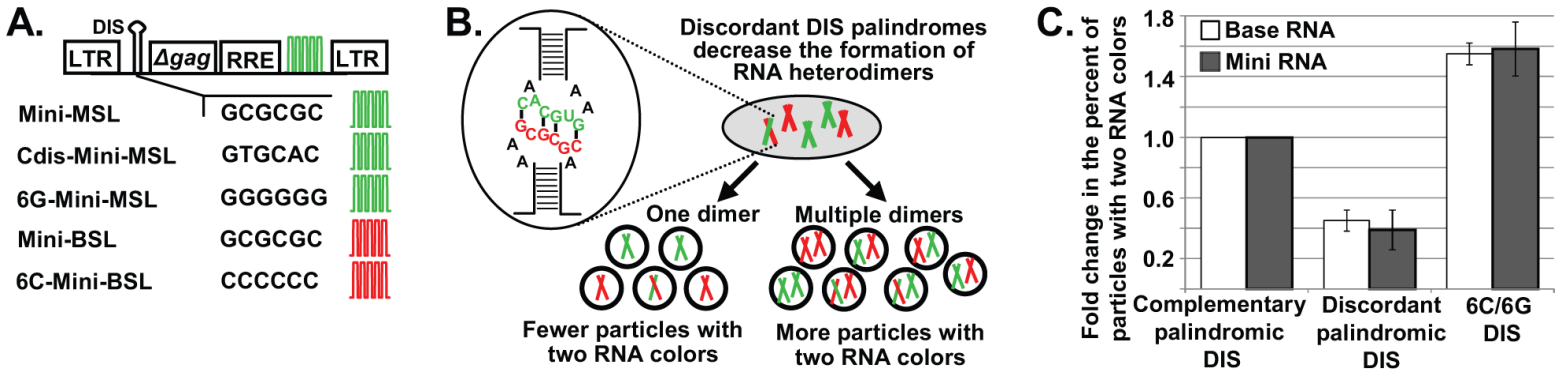
The regulation of short HIV-1 RNA packaging. (A) General structures of Mini HIV-1 constructs. The first 200 nt of *gag* is shown as Δ*gag*; RRE, Rev-response element; DIS, dimerization initiation signal. Other abbreviations are the same as in Figure 1. (B) Schematic representation of predicted outcomes when Mini RNAs are packaged as one dimer or more than one dimer with two constructs containing discordant DIS palindromes. (C) Effects of DIS sequences on the generation of two-RNA-color (YFP^+^mCherry^+^) particles containing Base (white bars) and Mini (gray bars) RNAs. The ratio of YFP^+^mCherry^+^ particles from two constructs that both contain GCGCGC in their DIS was set as 1. Data for Base RNAs were recalculated from data described in [16]. Values for Mini RNAs represent the mean ± SD from 5 independent experiments.

The “RNA mass” hypothesis proposes that a certain mass of viral RNA is packaged into each particle. Therefore, this hypothesis predicts that when the HIV-1 full-length RNA is much shorter than the wild-type HIV-1 genome, more than two copies of RNA should be encapsidated into one virion. To test this hypothesis, we generated Mini HIV-1 constructs that express ≈3-kb viral RNAs containing elements essential for the expression and packaging of the RNA, including an intact 5′ untranslated region, the first 200 nt of *gag*, the Rev-response element, and sequences recognized by the MS2 or BglG RNA-binding proteins (Figure 3A). To test whether Mini RNAs are packaged efficiently by HIV-1 Gag, we coexpressed Mini-MSL or Mini-BSL with helper constructs that express Gag, Gag-CeFP, Tat, Rev, and MS2-YFP and Bgl-mCherry. We found that ≈70%–80% of the CeFP^+^ particles were mCherry^+^ or YFP^+^ (5 independent experiments, >14,000 particles analyzed for each construct; Table S3), indicating that Mini RNAs were packaged into viral particles, albeit at an efficiency lower than that of Base RNA.

The ability of two HIV-1 RNAs to be copackaged into the same virus is strongly influenced by their DIS sequences. We have shown that RNAs from two Base vectors can be copackaged at a near-random level when both RNAs have GCGCGC in their DIS, whereas copackaging is decreased 2.3-fold when the two RNAs contain discordant DIS palindromes, one with GCGCGC and the other with GUGCAC [16], [17]. GCGCGC and GUGCAC are the DIS sequences in most subtype B and subtype C HIV-1, respectively [22]. We used this feature of HIV-1 biology to examine the number of Mini RNAs encapsidated into HIV-1 particles by single-virion analysis. We reasoned that if one Mini RNA dimer is packaged into a viral particle, then the inefficient heterodimer formation will be directly reflected in the ratio of particles with two RNA colors (YFP^+^mCherry^+^) (Figure 3B). Therefore, compared with two Mini RNAs that both contain GCGCGC in their DIS, Mini RNAs containing discordant palindromes (one with GCGCGC and another with GUGCAC) should have a reduced ratio of viral particles with two RNA colors; furthermore, the level of reduction should be similar to that of the Base vectors. In contrast, if multiple dimers are packaged into a viral particle, then the effects of reducing RNA heterodimers on the generation of particles with two RNA colors will be lessened, because many particles will contain one dimer in which both RNAs have GCGCGC and one dimer in which both RNAs have GUGCAC, thereby increasing the proportion of particles with two RNA colors (Figure 3B).

To test the effects of DIS sequences on the formation of particles with two RNA colors, we coexpressed Mini-MSL and Mini-BSL, both of which have GCGCGC in their DIS, along with helper constructs; ≈25.7% of the CeFP^+^ particles exhibited both mCherry and YFP signals. When we coexpressed Mini-BSL with Cdis-Mini-MSL that contains GUGCAC in its DIS, we found that ≈9.6% of the CeFP^+^ particles exhibited both RNA signals (YFP^+^mCherry^+^), which corresponds to a 2.7-fold reduction (25.7%/9.6%) from that produced by two Mini RNAs with GCGCGC in their DIS. We also examined the effects of changing the DIS sequences of Mini constructs into two complementary nonpalindromic sequences and generated 6G-Mini-MSL and 6C-Mini-BSL that contain GGGGGG and CCCCCC in their DISs, respectively. When coexpressed with helper constructs, 6G-Mini-MSL and 6C-Mini-BSL produced ≈40.4% of heterozygous particles, which corresponds to a 1.6-fold increase from that generated by Mini-MSL and Mini-BSL (5 experiments, >15,000 particles analyzed for each set of constructs; Table S4).

The effects of altering DIS sequences in Base vectors and in Mini vectors on the production of YFP^+^mCherry^+^ particles among CeFP^+^ particles are shown in Figure 3C. In these comparisons, the ratio of the heterozygous particles produced from two RNAs with GCGCGC in their DIS was set to 1 and the effects of changing the DIS sequence to discordant palindromes or complementary nonpalindromes are shown relative to that of vectors with GCGCGC. These comparisons revealed that changing DIS sequences influenced the ratios of heterozygous particles at the same levels in both Base and Mini constructs. Therefore, these results indicate that, even when HIV-1 genomic RNAs were much shorter than wild type (≈3 kb versus ≈9 kb), they were still encapsidated as one dimer and not as multiple dimers.

Taken together, these results show that HIV-1 particles have the capacity to accommodate HIV-1 RNA significantly larger than the 9-kb wild-type genome. However, regardless of whether the RNA was ≈17 kb or ≈3 kb, one dimer was packaged into a viral particle. These findings do not support the “RNA mass” packaging hypothesis; rather, they are consistent with the hypothesis that HIV-1 regulates its genome encapsidation by copy number.

### Single RNAs that Self-Dimerize Are Packaged

Our results from constructs with large RNA or Mini RNAs are consistent with the hypothesis that HIV-1 regulates genome packaging by RNA copy number. It is possible that HIV-1 regulates genome packaging by encapsidating one RNA dimer; alternatively, HIV-1 may exert the regulation by packaging two copies of viral RNA. We sought to determine the unit (i.e., one dimeric RNA or two monomeric RNAs) that HIV-1 uses to regulate RNA packaging. Sakuragi and colleagues previously demonstrated that by inserting into the *env* gene a second copy of the 5′ leader sequence including the DIS, the resulting RNAs can form both intermolecular dimers and intramolecular dimers (self-dimers) [32]. The intermolecular dimer and self-dimer are distinguished by using nondenaturing Northern analyses; the intermolecular dimer is a complex of two RNAs, whereas the self-dimer contains one RNA that migrates to a position expected for a monomeric RNA. In the experiments by Sakuragi et al., ≈60% and ≈40% of the virion RNAs were intermolecular and self-dimers, respectively [32].

**Figure 4:**
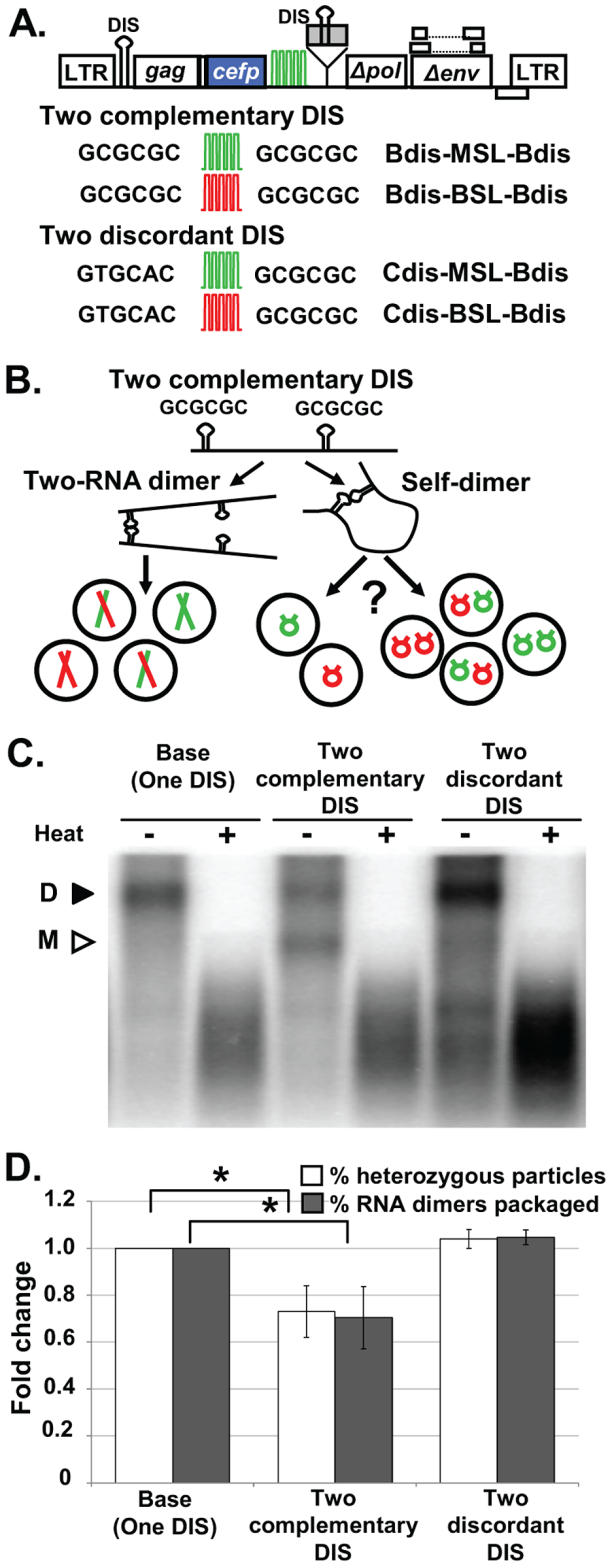
Encapsidation of HIV-1 RNAs containing two packaging/dimerization signals. (A) General structures of HIV-1 constructs containing two packaging signals. DIS sequences are shown; the gray box denotes the additional packaging signal inserted in *pol*. Other abbreviations are the same as in Figure 1. (B) Schematic representation of intermolecular and intramolecular dimers generated by RNAs containing two dimerization signals. (C) Representative nondenaturing Northern analyses of virion RNAs. Black and white arrowheads indicate the positions of RNA dimers and monomers, respectively. (D) Correlation between intermolecular dimer packaging and ratios of heterozygous particles. Gray bars, ratios of intermolecular dimers among the virion RNA samples determined by nondenaturing Northern analyses; white bars, ratios of heterozygous particles in the viral population determined by single-virion analyses. Values for the Base construct were set as 1; values for Northern analyses represent the mean ± SD from ≥2 experiments; values for single-virion analyses represent the mean ± SD from ≥9 experiments. Asterisks denote statistical significance (*P*<0.05; Student's t-test).

We used this strategy and generated HIV-1 vectors containing two packaging signals (Figure 4A) by inserting into the *pol* gene of the Base vectors a segment of the NL4-3 genome from U5 to the first 34 nt of *gag*, with a mutation in the splice donor site to abolish its function [32]. We generated viruses derived from these vectors containing two identical dimerization signals and analyzed the virion RNAs by nondenaturing Northern analyses. A representative nondenaturing Northern analysis is shown in Figure 4C. Before heat treatment, most of the virion RNAs from the parental Base vectors were dimers (94%; mean from 6 experiments); in contrast, virion RNAs from vectors with two identical dimerization signals contained both an intermolecular dimer and a faster-migrating population consistent with monomeric RNAs. The mean of the intermolecular dimers in 6 experiments was 66%, similar to that reported by Sakuragi and colleagues [32]. However, these Northern analyses results cannot address whether one or two copies of self-dimers are packaged in one particle; thus, we performed single-virion analyses to address this issue.

If HIV-1 regulates genome encapsidation by packaging two copies of RNAs, then two copies of the self-dimer should be packaged, and the ratio of YFP^+^mCherry^+^ particles should remain the same as that generated by the Base vectors. In contrast, if HIV-1 regulates the virion genome by packaging one dimeric RNA, then one copy of the self-dimer should be packaged, and the ratio of YFP^+^mCherry^+^ particles should be lower than that generated by the Base vectors (Figure 4B). We examined particles produced from the coexpression of constructs in which the DIS sequence in both packaging signals are from subtype B (GCGCGC), Bdis-BSL-Bdis and Bdis-MSL-Bdis, and found that the YFP^+^mCherry^+^ particle ratio in the viral population was reduced from ≈44% to ≈32% (Table S2 and S5). Our Northern analyses showed that approximately 66% of the virion RNAs were intermolecular dimers, assuming equal expression and random copackaging, half of these viruses should be heterozygous particles (≈33%). If one copy of the self-dimer was packaged, these particles will only confer one RNA color and will not increase the ratio of heterozygous particles; hence, we would expect that ≈33% of the virions would be YFP^+^ mCherry^+^ particles. Therefore, the level of reduction in heterozygous particles was consistent with the theoretical prediction based on one copy of the self-dimer being packaged (Figure 4D). To exclude the possibility that the duplicated HIV-1 sequences containing the second DIS affected RNA packaging, we generated and examined additional constructs containing discordant DIS palindromes. These constructs, Cdis-MSL-Bdis and Cdis-BSL-Bdis, contain GUGCAC in their DIS at the 5′ end of the viral RNA and GCGCGC in the second DIS within the sequences inserted in the *pol* gene (Figure 4A). As the two DIS sequences within the RNA are discordant palindromes, we expected that self-dimer formation would be discouraged and RNAs derived from these constructs would preferentially form intermolecular dimers. If so, these constructs would generate heterozygous particles efficiently. Nondenaturing Northern analyses demonstrated that most of the RNAs (98%) migrated to the position of the intermolecular dimer (Figure 4C). Single-virion analyses showed that coexpression of Cdis-MSL-Bdis and Cdis-BSL-Bdis produced heterozygous particles at a level similar to that of Base-MSL and Base-BSL viruses (Figure 4D and Table S5). These results indicate that when HIV-1 RNAs form self-dimers, only one copy of RNA is packaged. Therefore, HIV-1 can bypass the requirement for packaging two copies of RNA by recognizing a single copy of RNA containing a dimeric structure. Taken together, these findings suggest that HIV-1 regulates RNA packaging by recognizing a dimeric RNA, which leads to the packaging of two copies of the wild-type, full-length viral genome.

## Discussion

One of the essential steps in generating an infectious retroviral virion is the packaging of the RNA genome. The mechanism that regulates RNA packaging has many implications for retroviral evolution. For example, if a virion cannot accommodate two copies of full-length genomes exceeding a certain size, then this limitation would be a major factor in shaping the economical organization of the viral genome. Additionally, the mechanism that regulates RNA packaging and heterozygosity can affect genome diversity by altering the potential for recombination. Recombination occurs when reverse transcriptase copies genetic information from different portions of the two copackaged viral RNAs. If a certain mass of RNA is packaged, then multiple copies of smaller RNAs, or one large viral RNA, would be packaged into one virion; therefore, the size of the viral genome would directly affect the recombination potential of the virus. In this report, we address the long-standing question of how a retrovirus regulates the number of viral genomes packaged into each virion and show that HIV-1 regulates its RNA encapsidation not by the mass of the viral genome, but by packaging two copies of RNAs based on the recognition of an RNA dimer structure.

A major mechanism for retroviral oncogene transduction is the packaging of read-through transcripts followed by recombination [33], [34], [35]. The read-through transcripts are often significantly larger than the full-length genome, demonstrating that retroviruses can package RNAs larger than their genomes. However, the encapsidation efficiency of these read-through RNAs and whether one or two copies of the RNAs are packaged remains unknown. In this study, we found that most of the particles derived from XLong constructs with RNA close to 17 kb contain two copies of viral genomes; furthermore, EM analyses showed that the size and morphology of these particles are indistinguishable from those containing RNA genomes that are less than 9 kb. Therefore, immature HIV-1 particles can accommodate two copies of genomes larger than 9 kb. Despite its ability to package larger RNAs, HIV-1 uses overlapping open reading frames and splicing, rather than expanding its genome size, to encode all the genes that are required for efficient replication. We speculate that a larger genome has other fitness costs, such as inefficient expression, lower RNA stability, and reduced efficiency in completing reverse transcription, nuclear import, or integration, some of which were observed in another retrovirus [29]; the collective fitness burden associated with these factors may limit the ability of HIV-1 to expand its genome.

We have shown that HIV-1 regulates RNA packaging by recognizing a dimeric RNA structure. Specifically, particles packaging ≈3-kb Mini RNAs still contained only one dimer, not multiple dimers. Furthermore, RNAs containing two packaging signals that form self-dimers were encapsidated into HIV-1 particles as a single copy of the genome. How does HIV-1 package one dimer and not multiple dimers? We hypothesize that Gag-dimeric RNA interaction is the nucleation point of HIV-1 virus assembly, and that this interaction promotes the association of other Gag proteins leading to the formation of the virion. Because only one nucleation point is required to promote Gag recruitment, the dynamics of the virus assembly leads to the packaging of one dimer.

The molecular mechanism of preferential packaging of dimeric RNAs is well-established for murine leukemia virus. It was shown that RNA dimerization causes conformational changes and exposes high affinity nucleocapsid (NC) binding sites that are buried in the monomeric RNA [36]; furthermore, mutations of these binding sites led to significant decrease in the levels of RNA genome packaging [37]. A recent study shows that in HIV-1 dimeric RNA, interaction occurs between U5 and a region near the Gag translation start codon that leads to increases in NC binding [38]. Hence, using the differences in Gag's affinities to bind to dimeric versus monomeric viral RNA may be a general mechanism used by many retroviruses to ensure the packaging of dimeric RNA. As shown in this and other studies, the DIS plays an important role in the initiation of RNA dimerization and the selection of the copackaged RNA partner. However, the DIS is not the only RNA element in the viral genome that directs RNA dimerization as the virion RNAs from a DIS-deletion mutant are still dimeric [5], [6], [39]. Therefore, other currently unknown RNA elements must play a role in RNA dimerization as well.

Retroviruses are known to package an assortment of cellular mRNA into their particles, especially in the absence of the viral genome [40]. It is unclear whether the packaging of cellular mRNA is also regulated; if so, how the regulation is achieved. Additionally, we also do not know whether a nucleation point exists in the assembly of particles that lack viral RNA but contain cellular mRNA. It is possible that multiple lower-affinity Gag-nonviral RNA interaction events replace the nucleation point for virus assembly. Future studies are required to examine these questions.

Regulation of genome encapsidation has implications for multiple aspects of viral replication and evolution. Our results reveal that dimeric RNA recognition is the key element that regulates viral genome packaging into HIV-1 virions. These findings have direct implications for the dynamics of virus assembly, the potential for recombination to generate viral diversity, and the adaptive strategies employed by retroviruses for their replication.

## Methods

### Viral Vectors and Plasmids

For simplicity, previously described GagCeFP-BglSL and GagCeFP-MSSL [16] are referred to as Base-BSL and Base-MSL, respectively. Although only constructs expressing Gag tagged with Cerulean fluorescent protein (CeFP) are shown, each has a corresponding construct that expresses untagged Gag. Long-BSL was generated by first replacing a portion of *pol* that was deleted in Base-BSL, then inserting into the *nef* gene two DNA fragments, one from pB0-Spe6C [41] containing the mouse surface marker B7 gene and a mutated green fluorescent protein gene and another containing *lacZ*. The ouabain-resistance gene from pSVα3.6 [42] was cloned into Long-BSL to generate XLong-BSL. The AscI-to-SphI DNA fragments of Long-BSL and XLong-BSL plasmids were replaced with that from Base-MSL to generate Long-MSL and XLong-MSL plasmids, respectively.

Mini-MSL and Mini-BSL were derived from pKD-HIV(GFP-I-Hy) [43] by inserting sequences recognized by the MS2 coat protein or the BglG protein, respectively, in place of the sequences from the cytomegalovirus promoter to the end of hygromycin B phosphotransferase gene. Derivatives of Mini-MSL or Mini-BSL were generated by altering the DIS sequences. Bdis-BSL-Bdis and Bdis-MSL-Bdis were generated by inserting a PCR fragment containing nucleotides 555–823 (NL4-3 Genebank numbering) with a splice donor mutation from GGTG to GATC [32] into the *pol* gene of Base-BSL and Base-MSL, respectively. The 5′ DIS sequences of the two aforementioned plasmids were changed to GTGCAC to generate Cdis-BSL-Bdis and Cdis-MSL-Bdis. The structure of all plasmids was verified by restriction digests; PCR-amplified regions were confirmed by sequencing.

Plasmids expressing MS2-YFP and Bgl-mCherry have been reported previously [16], [44]. Helper constructs that were used to generate particles containing Mini RNAs include pSYNGP [45], which expresses codon-optimized HIV-1 Gag/GagPol; pSynGag-CeFP, which was generated by replacing *mCherry* in pSynGag-mCherry [46] with *cefp*; pTat-1 [47]; and pCMV-rev [48]. H0-PR* was derived from pON-H0 [49] with a D25N mutation in *pro*.

### Cell Culture, Virus Production, and Microscopy

Human 293T cells were maintained as previously described [16]. Transfections were performed using poly(ethylenimine) (PEI) reagent [15], FuGeneHD (Roche), or TransIT-LT1 (Muris). Supernatants were harvested 19–24 h post-transfection, clarified through a 0.45-µm-pore-size filter to remove cellular debris, and either stored at −80°C or used immediately. Fluorescence microscopy used in single-virion analyses [16] and electron microscopy (EM) analyses [50] were performed as previously described.

### Biochemical Analyses of Virion RNA

Viral particles were treated with RNase-free DNase to remove DNA prior to virion RNA isolation. Virion RNA isolation [51] and nondenaturing Northern blots were performed as previously described using riboprobes generated from a *gag* fragment [17]. Signal intensity was quantified using a phosphorimager. For denaturing Northern analyses, virion RNA isolation and hybridization were performed as previously described using a random-primed, ^32^P-labeled probe generated from an 8-kb AvaI digestion fragment that covered most of the NL4-3 sequences [52], [53]. RNA samples used in velocity gradients were isolated from virions pelleted through a 20% sucrose cushion, treated with proteinase K, and extracted with phenol/chloroform. Wild-type HIV-1 (NL4-3) RNA analyzed by velocity sedimentation was obtained from virus stocks prepared as described [54]. Total cellular RNA was prepared from 293T cells using Trizol reagent (Invitrogen). RNA samples were adjusted to contain 1% SDS (w/v) and 300 mM NaCl, and the cellular RNA samples were heat-denatured (83°C for 3 min) before loading on the gradient.

Linear 15%–30% sucrose (w/v) gradients were prepared as previously described [55] and centrifuged at 234,000×*g* for 80 or 120 min at 23°C in a Beckman SW41 Ti rotor. Collected fractions were analyzed by quantitative real-time RT-PCR. HIV-1 RNAs were detected using primers and probe sets annealed to *gag* as previously described [56]; human 28S rRNAs were detected using a primer and probe set from Roche Assay ID140876.

## Supporting Information

Figure S1 **EM analysis of HIV-1 particles produced from different viral constructs.** (A) Representative images of HIV-1 particles produced from different viral constructs. NL4-3 PR*, protease-deficient NL4-3-based construct H0-PR*. (B) Diameters of HIV-1 particles produced from different viral constructs. For each sample, at least 250 particles were measured. Values represent the mean ± SD from two independent experiments.
(DOC)

Table S1 **RNA contents of HIV-1 particles containing different genome sizes.**
(DOC)

Table S2 **Proportion of heterozygous particles generated from constructs with different genome sizes.**
(DOC)

Table S3 **Proportions of HIV-1 particles containing Mini RNA genome.**
(DOC)

Table S4 **Effects of DIS sequences on the proportion of two-RNA-color viral particles containing Mini RNAs.** ^*^ Within each experiment, the percent of particles with two RNA colors for each sample was compared to the percent of particles with two RNA colors in the Mini-MSL + Mini-BSL sample.
(DOC)

Table S5 **Effects of DIS sequences on the percent of heterozygous particles containing HIV-1 RNAs with two dimerization signals.** ^*^ The percent of heterozygous particles in each sample was compared to the average percent of heterozygous particles for Base-MSL + Base-BSL samples in Supplemental table S2 that was set to 1.0.
(DOC)

## References

[1] Temin HM (1976) The DNA provirus hypothesis. Science 192: 1075–1080. 58444 10.1126/science.58444

[2] Duesberg PH (1968) Physical properties of Rous Sarcoma Virus RNA. Proc Natl Acad Sci U S A 60: 1511–1518. 4299948 10.1073/pnas.60.4.1511 PMC224948

[3] Kung HJ, Hu S, Bender W, Bailey JM, Davidson N, et al (1976) RD-114, baboon, and woolly monkey viral RNA's compared in size and structure. Cell 7: 609–620. 182377 10.1016/0092-8674(76)90211-7

[4] Fu W, Gorelick RJ, Rein A (1994) Characterization of human immunodeficiency virus type 1 dimeric RNA from wild-type and protease-defective virions. J Virol 68: 5013–5018. 8035501 10.1128/jvi.68.8.5013-5018.1994 PMC236443

[5] Paillart JC, Shehu-Xhilaga M, Marquet R, Mak J (2004) Dimerization of retroviral RNA genomes: an inseparable pair. Nat Rev Microbiol 2: 461–472. 15152202 10.1038/nrmicro903

[6] Moore MD, Hu WS (2009) HIV-1 RNA dimerization: It takes two to tango. AIDS Rev 11: 91–102. 19529749 PMC3056336

[7] Luban J, Goff SP (1991) Binding of human immunodeficiency virus type 1 (HIV-1) RNA to recombinant HIV-1 gag polyprotein. Journal of virology 65: 3203–3212. 2033671 10.1128/jvi.65.6.3203-3212.1991 PMC240977

[8] Aldovini A, Young RA (1990) Mutations of RNA and protein sequences involved in human immunodeficiency virus type 1 packaging result in production of noninfectious virus. Journal of virology 64: 1920–1926. 2109098 10.1128/jvi.64.5.1920-1926.1990 PMC249345

[9] Lever AM (2007) HIV-1 RNA packaging. Adv Pharmacol 55: 1–32. 17586311 10.1016/S1054-3589(07)55001-5

[10] Lever A, Gottlinger H, Haseltine W, Sodroski J (1989) Identification of a sequence required for efficient packaging of human immunodeficiency virus type 1 RNA into virions. Journal of virology 63: 4085–4087. 2760989 10.1128/jvi.63.9.4085-4087.1989 PMC251012

[11] McBride MS, Panganiban AT (1996) The human immunodeficiency virus type 1 encapsidation site is a multipartite RNA element composed of functional hairpin structures. J Virol 70: 2963–2973. 8627772 10.1128/jvi.70.5.2963-2973.1996 PMC190155

[12] Clever J, Sassetti C, Parslow TG (1995) RNA secondary structure and binding sites for gag gene products in the 5′ packaging signal of human immunodeficiency virus type 1. J Virol 69: 2101–2109. 7884856 10.1128/jvi.69.4.2101-2109.1995 PMC188876

[13] D'Souza V, Summers MF (2005) How retroviruses select their genomes. Nat Rev Microbiol 3: 643–655. 16064056 10.1038/nrmicro1210

[14] Wilkinson KA, Gorelick RJ, Vasa SM, Guex N, Rein A, et al (2008) High-throughput SHAPE analysis reveals structures in HIV-1 genomic RNA strongly conserved across distinct biological states. PLoS Biol 6: e96. 18447581 10.1371/journal.pbio.0060096 PMC2689691

[15] Moore MD, Nikolaitchik OA, Chen J, Hammarskjold ML, Rekosh D, et al (2009) Probing the HIV-1 genomic RNA trafficking pathway and dimerization by genetic recombination and single virion analyses. PLoS Pathog 5: e1000627. 19834549 10.1371/journal.ppat.1000627 PMC2757677

[16] Chen J, Nikolaitchik O, Singh J, Wright A, Bencsics CE, et al (2009) High efficiency of HIV-1 genomic RNA packaging and heterozygote formation revealed by single virion analysis. Proc Natl Acad Sci U S A 106: 13535–13540. 19628694 10.1073/pnas.0906822106 PMC2714765

[17] Moore MD, Fu W, Nikolaitchik O, Chen J, Ptak RG, et al (2007) Dimer initiation signal of human immunodeficiency virus type 1: its role in partner selection during RNA copackaging and its effects on recombination. J Virol 81: 4002–4011. 17267488 10.1128/JVI.02589-06 PMC1866129

[18] Laughrea M, Jette L (1996) Kissing-loop model of HIV-1 genome dimerization: HIV-1 RNAs can assume alternative dimeric forms, and all sequences upstream or downstream of hairpin 248–271 are dispensable for dimer formation. Biochemistry 35: 1589–1598. 8634290 10.1021/bi951838f

[19] Muriaux D, Fosse P, Paoletti J (1996) A kissing complex together with a stable dimer is involved in the HIV-1Lai RNA dimerization process in vitro. Biochemistry 35: 5075–5082. 8664300 10.1021/bi952822s

[20] Paillart JC, Skripkin E, Ehresmann B, Ehresmann C, Marquet R (1996) A loop-loop “kissing” complex is the essential part of the dimer linkage of genomic HIV-1 RNA. Proc Natl Acad Sci U S A 93: 5572–5577. 8643617 10.1073/pnas.93.11.5572 PMC39288

[21] Clever JL, Wong ML, Parslow TG (1996) Requirements for kissing-loop-mediated dimerization of human immunodeficiency virus RNA. J Virol 70: 5902–5908. 8709210 10.1128/jvi.70.9.5902-5908.1996 PMC190608

[22] Hussein IT, Ni N, Galli A, Chen J, Moore MD, et al (2010) Delineation of the preferences and requirements of the human immunodeficiency virus type 1 dimerization initiation signal by using an in vivo cell-based selection approach. J Virol 84: 6866–6875. 20410279 10.1128/JVI.01930-09 PMC2903283

[23] St Louis DC, Gotte D, Sanders-Buell E, Ritchey DW, Salminen MO, et al (1998) Infectious molecular clones with the nonhomologous dimer initiation sequences found in different subtypes of human immunodeficiency virus type 1 can recombine and initiate a spreading infection in vitro. J Virol 72: 3991–3998. 9557686 10.1128/jvi.72.5.3991-3998.1998 PMC109626

[24] Sakalian M, Wills JW, Vogt VM (1994) Efficiency and selectivity of RNA packaging by Rous sarcoma virus Gag deletion mutants. Journal of virology 68: 5969–5981. 8057473 10.1128/jvi.68.9.5969-5981.1994 PMC237002

[25] Vogt VM (1997) Retroviral Virions and Genomes. In: Coffin JM, Hughes, S.H, Varmus, H.E., editor. Retroviruses. Cold Spring Harbor: Cold Spring Harbor Laboratory Press. pp. 27–69. 21433348

[26] Gelinas C, Temin HM (1986) Nondefective spleen necrosis virus-derived vectors define the upper size limit for packaging reticuloendotheliosis viruses. Proceedings of the National Academy of Sciences of the United States of America 83: 9211–9215. 3024171 10.1073/pnas.83.23.9211 PMC387105

[27] Terwilliger EF, Godin B, Sodroski JG, Haseltine WA (1989) Construction and use of a replication-competent human immunodeficiency virus (HIV-1) that expresses the chloramphenicol acetyltransferase enzyme. Proceedings of the National Academy of Sciences of the United States of America 86: 3857–3861. 2726755 10.1073/pnas.86.10.3857 PMC287240

[28] Swanstrom R, and Wills J.W. (1997) Synthesis, Assembly, and Processing of Viral Proteins. In: Coffin JM, Hughes, S.H, Varmus, H.E., editor. Retroviruses. Cold Spring Harbor: Cold Spring Harbor Press. pp. 263–334. 21433349

[29] Shin NH, Hartigan-O'Connor D, Pfeiffer JK, Telesnitsky A (2000) Replication of lengthened Moloney murine leukemia virus genomes is impaired at multiple stages. Journal of virology 74: 2694–2702. 10684285 10.1128/jvi.74.6.2694-2702.2000 PMC111759

[30] Kumar M, Keller B, Makalou N, Sutton RE (2001) Systematic determination of the packaging limit of lentiviral vectors. Hum Gene Ther 12: 1893–1905. 11589831 10.1089/104303401753153947

[31] Larson DR, Johnson MC, Webb WW, Vogt VM (2005) Visualization of retrovirus budding with correlated light and electron microscopy. Proc Natl Acad Sci U S A 102: 15453–15458. 16230638 10.1073/pnas.0504812102 PMC1266096

[32] Sakuragi J, Shioda T, Panganiban AT (2001) Duplication of the primary encapsidation and dimer linkage region of human immunodeficiency virus type 1 RNA results in the appearance of monomeric RNA in virions. J Virol 75: 2557–2565. 11222678 10.1128/JVI.75.6.2557-2565.2001 PMC115878

[33] Swain A, Coffin JM (1992) Mechanism of transduction by retroviruses. Science 255: 841–845. 1371365 10.1126/science.1371365

[34] Goff SP, Gilboa E, Witte ON, Baltimore D (1980) Structure of the Abelson murine leukemia virus genome and the homologous cellular gene: studies with cloned viral DNA. Cell 22: 777–785. 6257398 10.1016/0092-8674(80)90554-1

[35] Herman SA, Coffin JM (1987) Efficient packaging of readthrough RNA in ALV: implications for oncogene transduction. Science 236: 845–848. 3033828 10.1126/science.3033828

[36] D'Souza V, Summers MF (2004) Structural basis for packaging the dimeric genome of Moloney murine leukaemia virus. Nature 431: 586–590. 15457265 10.1038/nature02944

[37] Gherghe C, Lombo T, Leonard CW, Datta SA, Bess JW Jr, et al (2010) Definition of a high-affinity Gag recognition structure mediating packaging of a retroviral RNA genome. Proc Natl Acad Sci U S A 107: 19248–19253. 20974908 10.1073/pnas.1006897107 PMC2984214

[38] Lu K, Heng X, Garyu L, Monti S, Garcia EL, et al (2011) NMR detection of structures in the HIV-1 5′-leader RNA that regulate genome packaging. Science 334: 242–245. 21998393 10.1126/science.1210460 PMC3335204

[39] Hill MK, Shehu-Xhilaga M, Campbell SM, Poumbourios P, Crowe SM, et al (2003) The dimer initiation sequence stem-loop of human immunodeficiency virus type 1 is dispensable for viral replication in peripheral blood mononuclear cells. J Virol 77: 8329–8335. 12857902 10.1128/JVI.77.15.8329-8335.2003 PMC165254

[40] Rulli SJ Jr, Hibbert CS, Mirro J, Pederson T, Biswal S, et al (2007) Selective and nonselective packaging of cellular RNAs in retrovirus particles. J Virol 81: 6623–6631. 17392359 10.1128/JVI.02833-06 PMC1900105

[41] Nikolaitchik OA, Galli A, Moore MD, Pathak VK, Hu WS (2011) Multiple barriers to recombination between divergent HIV-1 variants revealed by a dual-marker recombination assay. Journal of molecular biology 407: 521–531. 21295586 10.1016/j.jmb.2011.01.052 PMC3065980

[42] Kent RB, Emanuel JR, Ben Neriah Y, Levenson R, Housman DE (1987) Ouabain resistance conferred by expression of the cDNA for a murine Na+, K+-ATPase alpha subunit. Science 237: 901–903. 3039660 10.1126/science.3039660

[43] Nikolenko GN, Svarovskaia ES, Delviks KA, Pathak VK (2004) Antiretroviral drug resistance mutations in human immunodeficiency virus type 1 reverse transcriptase increase template-switching frequency. J Virol 78: 8761–8770. 15280484 10.1128/JVI.78.16.8761-8770.2004 PMC479068

[44] Fusco D, Accornero N, Lavoie B, Shenoy SM, Blanchard JM, et al (2003) Single mRNA molecules demonstrate probabilistic movement in living mammalian cells. Curr Biol 13: 161–167. 12546792 10.1016/s0960-9822(02)01436-7 PMC4764064

[45] Kotsopoulou E, Kim VN, Kingsman AJ, Kingsman SM, Mitrophanous KA (2000) A Rev-independent human immunodeficiency virus type 1 (HIV-1)-based vector that exploits a codon-optimized HIV-1 gag-pol gene. J Virol 74: 4839–4852. 10775623 10.1128/jvi.74.10.4839-4852.2000 PMC112007

[46] Burdick R, Smith JL, Chaipan C, Friew Y, Chen J, et al (2010) P body-associated protein Mov10 inhibits HIV-1 replication at multiple stages. Journal of virology 84: 10241–10253. 20668078 10.1128/JVI.00585-10 PMC2937795

[47] Peterlin BM, Luciw PA, Barr PJ, Walker MD (1986) Elevated levels of mRNA can account for the trans-activation of human immunodeficiency virus. Proceedings of the National Academy of Sciences of the United States of America 83: 9734–9738. 3025848 10.1073/pnas.83.24.9734 PMC387215

[48] Lewis N, Williams J, Rekosh D, Hammarskjold ML (1990) Identification of a cis-acting element in human immunodeficiency virus type 2 (HIV-2) that is responsive to the HIV-1 rev and human T-cell leukemia virus types I and II rex proteins. Journal of virology 64: 1690–1697. 2157051 10.1128/jvi.64.4.1690-1697.1990 PMC249306

[49] Rhodes TD, Nikolaitchik O, Chen J, Powell D, Hu WS (2005) Genetic recombination of human immunodeficiency virus type 1 in one round of viral replication: effects of genetic distance, target cells, accessory genes, and lack of high negative interference in crossover events. J Virol 79: 1666–1677. 15650192 10.1128/JVI.79.3.1666-1677.2005 PMC544095

[50] Tobin GJ, Nagashima K, Gonda MA (1996) Immunologic and Ultrastructural Characterization of HIV Pseudovirions Containing Gag and Env Precursor Proteins Engineered in Insect Cells. Methods 10: 208–218. 8812671 10.1006/meth.1996.0096

[51] Fu W, Rein A (1993) Maturation of dimeric viral RNA of Moloney murine leukemia virus. J Virol 67: 5443–5449. 8350405 10.1128/jvi.67.9.5443-5449.1993 PMC237946

[52] Gorelick RJ, Benveniste RE, Gagliardi TD, Wiltrout TA, Busch LK, et al (1999) Nucleocapsid protein zinc-finger mutants of simian immunodeficiency virus strain mne produce virions that are replication defective in vitro and in vivo. Virology 253: 259–270. 9918884 10.1006/viro.1998.9513

[53] Gorelick RJ, Nigida SM Jr, Bess JW Jr, Arthur LO, Henderson LE, et al (1990) Noninfectious human immunodeficiency virus type 1 mutants deficient in genomic RNA. Journal of virology 64: 3207–3211. 2191147 10.1128/jvi.64.7.3207-3211.1990 PMC249531

[54] Watts JM, Dang KK, Gorelick RJ, Leonard CW, Bess JW Jr, et al (2009) Architecture and secondary structure of an entire HIV-1 RNA genome. Nature 460: 711–716. 19661910 10.1038/nature08237 PMC2724670

[55] Cheevers WP, Archer BG, Crawford TB (1977) Characterization of RNA from equine infectious anemia virus. Journal of virology 24: 489–497. 199735 10.1128/jvi.24.2.489-497.1977 PMC515958

[56] Buckman JS, Bosche WJ, Gorelick RJ (2003) Human immunodeficiency virus type 1 nucleocapsid zn(2+) fingers are required for efficient reverse transcription, initial integration processes, and protection of newly synthesized viral DNA. J Virol 77: 1469–1480. 12502862 10.1128/JVI.77.2.1469-1480.2003 PMC140799

