# Supplementary material for: Dimeric RNA Recognition Regulates HIV-1 Genome Packaging
Source: PLoS Pathog. 2013 Mar 21;9(3):e1003249. doi: 10.1371/journal.ppat.1003249 (PMC3605237; doi:10.1371/journal.ppat.1003249)
Supplement: Figure S1 — EM analysis of HIV-1 particles produced from different viral constructs. (A) Representative images of HIV-1 particles produced from different viral constructs. NL4-3 PR*, protease-deficient NL4-3-based construct H0-PR*. (B) Diameters of HIV-1 particles produced from different viral constructs. For each sample, at least 250 particles were measured. Values represent the mean ± SD from two independent experiments. (DOC) [file ppat.1003249.s001.doc]

**Figure S1**. EM analysis of HIV-1 particles produced from different viral constructs. (A) Representative images of HIV-1 particles produced from different viral constructs. NL4-3 PR*, protease-deficient NL4-3-based construct H0-PR*. (B) Diameters of HIV-1 particles produced from different viral constructs. For each sample, at least 250 particles were measured. Values represent the mean ± SD from two independent experiments.
