# Supplementary material for: Dimeric RNA Recognition Regulates HIV-1 Genome Packaging
Source: PLoS Pathog. 2013 Mar 21;9(3):e1003249. doi: 10.1371/journal.ppat.1003249 (PMC3605237; doi:10.1371/journal.ppat.1003249)
Supplement: Table S1 — RNA contents of HIV-1 particles containing different genome sizes. (DOC) [file ppat.1003249.s002.doc]

**Table S1. RNA contents of HIV-1 particles containing different genome sizes.**

| **Constructs** | **Number of particles analyzed** | **CeFP^+^ YFP^+^(%)** | **CeFP^+^ mCherry^+^ (%)** | **CeFP^+^ YFP^+^ mCherry^+^ (%)** | **RNA labeling efficiency(%)** |
| --- | --- | --- | --- | --- | --- |
| **Base-MSL** | | |  |  |  |
| Exp 1 | 2706 | 94.8 | 0.0 | 0.0 | 94.8 |
| Exp 2 | 1131 | 93.7 | 0.0 | 0.0 | 93.7 |
| Exp 3 | 6511 | 90.0 | 0.0 | 0.2 | 90.2 |
| Exp 4 | 2923 | 96.0 | 0.0 | 0.1 | 96.1 |
| Exp 5 | 2433 | 91.1 | 0.0 | 0.5 | 91.5 |
| Mean ± SD |  |  |  |  | 93.3 ± 2.4 |
|  |  |  |  |  |  |
| **Base-BSL** | | |  |  |  |
| Exp 1 | 2717 | 0.0 | 95.6 | 1.8 | 97.4 |
| Exp 2 | 1738 | 0.0 | 97.9 | 0.3 | 98.2 |
| Exp 3 | 5741 | 0.1 | 92.3 | 0.9 | 93.2 |
| Exp 4 | 3628 | 0.0 | 98.0 | 0.4 | 98.4 |
| Exp 5 | 4002 | 0.0 | 94.1 | 0.4 | 94.6 |
| Mean ± SD |  |  |  |  | 96.3 ± 2.3 |
|  |  |  |  |  |  |
| **Long-MSL** | | |  |  |  |
| Exp 1 | 1458 | 90.9 | 0.0 | 0.2 | 91.1 |
| Exp 2 | 642 | 95.3 | 0.0 | 0.0 | 95.3 |
| Exp 3 | 3799 | 93.3 | 0.0 | 0.1 | 93.4 |
| Exp 4 | 3218 | 92.4 | 0.0 | 0.2 | 92.7 |
| Mean ± SD |  |  |  |  | 93.1 ± 1.8 |
|  |  |  |  |  |  |
| **Long-BSL** | | |  |  |  |
| Exp 1 | 514 | 0.0 | 94.6 | 0.2 | 94.7 |
| Exp 2 | 2555 | 0.0 | 96.2 | 0.5 | 96.8 |
| Exp 3 | 9115 | 0.0 | 94.2 | 0.2 | 94.4 |
| Exp 4 | 10245 | 0.0 | 95.1 | 1.0 | 96.0 |
| Mean ± SD |  |  |  |  | 95.5 ± 1.1 |
|  |  |  |  |  |  |
| **XLong-MSL** | | |  |  |  |
| Exp 1 | 3661 | 91.9 | 0.0 | 0.3 | 92.2 |
| Exp 2 | 5532 | 92.6 | 0.0 | 0.4 | 93.0 |
| Exp 3 | 5730 | 93.9 | 0.0 | 0.2 | 94.1 |
| Exp 4 | 2703 | 92.3 | 0.0 | 0.1 | 92.5 |
| Mean ± SD |  |  |  |  | 92.9 ± 0.8 |
|  |  |  |  |  |  |
| **XLong-BSL** | | |  |  |  |
| Exp 1 | 3431 | 0.0 | 96.4 | 0.1 | 96.4 |
| Exp 2 | 6480 | 0.0 | 96.7 | 0.2 | 96.9 |
| Exp 3 | 10501 | 0.0 | 96.1 | 0.5 | 96.6 |
| Exp 4 | 5314 | 0.0 | 93.2 | 0.2 | 93.3 |
| Mean ± SD |  |  |  |  | 95.8 ± 1.7 |
