# Supplementary material for: Dimeric RNA Recognition Regulates HIV-1 Genome Packaging
Source: PLoS Pathog. 2013 Mar 21;9(3):e1003249. doi: 10.1371/journal.ppat.1003249 (PMC3605237; doi:10.1371/journal.ppat.1003249)
Supplement: Table S2 — Proportion of heterozygous particles generated from constructs with different genome sizes. (DOC) [file ppat.1003249.s003.doc]

**Table S2. Proportion of heterozygous particles generated from constructs with different genome sizes.**

| **Constructs** | **Number of particles analyzed** | **CeFP^+^ YFP^+^ (%)** | **CeFP^+^ mCherry^+^ (%)** | **CeFP^+^ YFP^+^ mCherry^+^ (%)** |
| --- | --- | --- | --- | --- |
| **Base-MSL + Base-BSL** | | |  |  |
| Exp 1 | 2770 | 21.0 | 29.1 | 45.8 |
| Exp 2 | 1374 | 26.0 | 27.6 | 43.5 |
| Exp 3 | 12129 | 20.1 | 30.3 | 42.0 |
| Exp 4 | 4112 | 32.1 | 19.1 | 46.3 |
| Exp 5 | 3158 | 28.4 | 24.0 | 42.0 |
| Mean ± SD |  |  |  | 43.9 ± 2.0 |
|  |  |  |  |  |
| **Long-MSL + Long-BSL** | | |  |  |
| Exp 1 | 4228 | 29.9 | 25.7 | 39.9 |
| Exp 2 | 10257 | 25.4 | 29.2 | 38.7 |
| Exp 3 | 6513 | 27.4 | 25.3 | 41.7 |
| Mean ± SD |  |  |  | 40.1 ± 1.5 |
|  |  |  |  |  |
| **XLong-MSL + XLong-BSL** | | |  |  |
| Exp 1 | 4832 | 20.7 | 35.1 | 40.1 |
| Exp 2 | 11899 | 23.8 | 31.0 | 38.5 |
| Exp 3 | 6961 | 25.5 | 29.7 | 37.7 |
| Exp 4 | 13158 | 32.8 | 23.8 | 36.8 |
| Mean ± SD |  |  |  | 38.3 ± 1.4 |
