# Supplementary material for: Dimeric RNA Recognition Regulates HIV-1 Genome Packaging
Source: PLoS Pathog. 2013 Mar 21;9(3):e1003249. doi: 10.1371/journal.ppat.1003249 (PMC3605237; doi:10.1371/journal.ppat.1003249)
Supplement: Table S3 — Proportions of HIV-1 particles containing Mini RNA genome. (DOC) [file ppat.1003249.s004.doc]

**Table S3. Proportions of HIV-1 particles containing Mini RNA genome.**

| **Constructs** | **Number of particles analyzed** | **CeFP^+^ YFP^+^ (%)** | **CeFP^+^ mCherry^+^ (%)** | **CeFP^+^ YFP^+^ mCherry^+^ (%)** | **RNA labeling efficiency (%)** |
| --- | --- | --- | --- | --- | --- |
| **Mini-MSL** | |  |  |  |  |
| Exp 1 | 1448 | 77.2 | 0.0 | 0.0 | 77.2 |
| Exp 2 | 1075 | 80.3 | 0.0 | 0.0 | 80.3 |
| Exp 3 | 2119 | 77.1 | 0.1 | 0.3 | 77.5 |
| Exp 4 | 8553 | 77.9 | 0.0 | 0.1 | 78.0 |
| Exp 5 | 1975 | 83.0 | 0.0 | 0.1 | 83.1 |
| Mean ± SD |  |  |  |  | 79.2 ± 2.5 |
|  |  |  |  |  |  |
| **Mini-BSL** |  |  |  |  |  |
| Exp 1 | 1056 | 0.7 | 70.4 | 1.0 | 72.1 |
| Exp 2 | 1426 | 0.1 | 87.5 | 0.4 | 88.1 |
| Exp 3 | 1813 | 0.2 | 65.6 | 0.4 | 66.2 |
| Exp 4 | 6132 | 0.0 | 79.5 | 0.3 | 79.8 |
| Exp 5 | 3587 | 0.1 | 84.0 | 0.1 | 84.2 |
| Mean ± SD |  |  |  |  | 78.1 ± 8.9 |
