# Supplementary material for: Dimeric RNA Recognition Regulates HIV-1 Genome Packaging
Source: PLoS Pathog. 2013 Mar 21;9(3):e1003249. doi: 10.1371/journal.ppat.1003249 (PMC3605237; doi:10.1371/journal.ppat.1003249)
Supplement: Table S4 — Effects of DIS sequences on the proportion of two-RNA-color viral particles containing Mini RNAs. * Within each experiment, the percent of particles with two RNA colors for each sample was compared to the percent of particles with two RNA colors in the Mini-MSL + Mini-BSL sample. (DOC) [file ppat.1003249.s005.doc]

**Table S4. Effects of DIS sequences on the proportion of two-RNA-color viral particles containing Mini RNAs.**

| **Constructs** | **Number of particles analyzed** | **CeFP^+^ YFP^+^ (%)** | **CeFP^+^ mCherry^+^ (%)** | **CeFP^+^ YFP^+^ mCherry^+^ (%)** | **Fold change in % of particles with two RNA colors*** |
| --- | --- | --- | --- | --- | --- |
| **Mini-MSL + Mini-BSL** | | |  |  |  |
| Exp 1 | 1672 | 37.9 | 10.7 | 22.4 | 1.0 |
| Exp 2 | 1824 | 23.9 | 32.0 | 27.1 | 1.0 |
| Exp 3 | 1351 | 21.9 | 24.9 | 19.5 | 1.0 |
| Exp 4 | 7360 | 21.9 | 31.2 | 28.6 | 1.0 |
| Exp 5 | 5922 | 30.7 | 25.1 | 30.9 | 1.0 |
| Mean ± SD |  |  |  | 25.7 ± 4.6 | 1.0 ± 0.0 |
|  |  |  |  |  |  |
| **Cdis-Mini-MSL + Mini-BSL** | | |  |  |  |
| Exp 1 | 4291 | 38.5 | 29.8 | 8.9 | 0.4 |
| Exp 2 | 2624 | 34.3 | 40.1 | 9.9 | 0.4 |
| Exp 3 | 2213 | 36.1 | 31.8 | 11.9 | 0.6 |
| Exp 4 | 5543 | 34.8 | 32.3 | 8.7 | 0.3 |
| Exp 5 | 4387 | 44.9 | 30.8 | 8.4 | 0.3 |
| Mean ± SD |  |  |  | 9.6 ± 1.4 | 0.4 ± 0.1 |
|  |  |  |  |  |  |
| **6G-Mini-MSL + 6C-Mini-BSL** | | |  |  |  |
| Exp 1 | 2068 | 22.2 | 10.8 | 36.7 | 1.6 |
| Exp 2 | 1096 | 18.0 | 16.5 | 45.1 | 1.7 |
| Exp 3 | 1852 | 32.8 | 8.5 | 35.7 | 1.8 |
| Exp 4 | 5929 | 10.7 | 20.7 | 39.0 | 1.4 |
| Exp 5 | 4696 | 15.0 | 16.2 | 45.7 | 1.5 |
| Mean ± SD |  |  |  | 40.4 ± 4.7 | 1.6 ± 0.2 |

^*^ Within each experiment, the percent of particles with two RNA colors for each sample was compared to the percent of particles with two RNA colors in the Mini-MSL + Mini-BSL sample.
