# Supplementary material for: Dimeric RNA Recognition Regulates HIV-1 Genome Packaging
Source: PLoS Pathog. 2013 Mar 21;9(3):e1003249. doi: 10.1371/journal.ppat.1003249 (PMC3605237; doi:10.1371/journal.ppat.1003249)
Supplement: Table S5 — Effects of DIS sequences on the percent of heterozygous particles containing HIV-1 RNAs with two dimerization signals. * The percent of heterozygous particles in each sample was compared to the average percent of heterozygous particles for Base-MSL + Base-BSL samples in Supplemental table S2 that was set to 1.0. (DOC) [file ppat.1003249.s006.doc]

**Table S5. Effects of DIS sequences on the percent of heterozygous particles containing HIV-1 RNAs with two dimerization signals.**

| **Sample** | **Number of particles analyzed** | **CeFP+ YFP+ (%)** | **CeFP+ mCherry+ (%)** | **CeFP+ YFP+ mCherry+ (%)** | **Fold change in % of heterozygous particles*** |
| --- | --- | --- | --- | --- | --- |
| **Bdis-MSL-Bdis + Bdis-BSL-Bdis** | |  |  |  |  |
| Exp 1 | 1121 | 28.7 | 30.1 | 33.4 | 0.8 |
| Exp 2 | 1343 | 34.8 | 31.0 | 24.3 | 0.6 |
| Exp 3 | 11403 | 16.0 | 48.5 | 25.6 | 0.6 |
| Exp 4 | 6494 | 23.9 | 30.7 | 36.4 | 0.8 |
| Exp 5 | 1429 | 34.7 | 27.4 | 31.6 | 0.7 |
| Exp 6 | 3071 | 26.6 | 37.8 | 30.0 | 0.7 |
| Exp 7 | 1458 | 37.4 | 24.6 | 28.2 | 0.6 |
| Exp 8 | 8521 | 27.8 | 25.3 | 37.7 | 0.9 |
| Exp 9 | 6427 | 39.4 | 19.2 | 36.5 | 0.8 |
| Exp 10 | 24477 | 31.9 | 22.3 | 36.5 | 0.8 |
| Mean ± SD |  |  |  | 32.0 ± 4.9 | 0.7 ± 0.1 |
|  |  |  |  |  |  |
| **Cdis-MSL-Bdis + Cdis-BSL-Bdis** | | |  |  |  |
| Exp 1 | 1512 | 34.3 | 17.5 | 46.4 | 1.1 |
| Exp 2 | 2809 | 19.4 | 32.5 | 44.9 | 1.0 |
| Exp 3 | 8582 | 21.6 | 25.5 | 47.2 | 1.1 |
| Exp 4 | 9609 | 17.5 | 30.6 | 46.7 | 1.1 |
| Exp 5 | 6394 | 25.5 | 29.0 | 42.2 | 1.0 |
| Exp 6 | 3146 | 20.0 | 28.5 | 46.8 | 1.1 |
| Exp 7 | 2846 | 24.4 | 27.8 | 43.1 | 1.0 |
| Exp 8 | 6812 | 20.8 | 27.6 | 47.2 | 1.1 |
| Exp 9 | 4770 | 32.3 | 18.9 | 45.3 | 1.0 |
| Mean ± SD |  |  |  | 45.5 ± 1.8 | 1.0 ± 0.0 |

^*^ The percent of heterozygous particles in each sample was compared to the average percent of heterozygous particles for Base-MSL + Base-BSL samples in Supplemental table S2 that was set to 1.0.
